# Supplementary material for: Payload distribution and capacity of mRNA lipid nanoparticles
Source: Nat Commun. 2022 Sep 23;13:5561. doi: 10.1038/s41467-022-33157-4 (PMC9508184; doi:10.1038/s41467-022-33157-4)
Supplement: Supplementary file 1 — Supplementary Information [file 41467_2022_33157_MOESM1_ESM.pdf]

**Supplementary Information**

# Payload Distribution and Capacity of mRNA Lipid Nanoparticles

Sixuan Li[1,#], Yizong Hu[2,3,4,#,*], Andrew Li[3], Jinghan Lin[2,3], Kuangwen Hsieh[1], Zachary Schneiderman[2,5], Pengfei Zhang[3], Yining Zhu[2,3,4], Chenhu Qiu[2,6], Efrosini Kokkoli[2,5], Tza-Huei Wang[1,2,3,*], and Hai-Quan Mao[2,3,4,6,*]

[1]Department of Mechanical Engineering, Johns Hopkins University, Baltimore, MD, USA. [2]Institute for NanoBioTechnology, Johns Hopkins University, Baltimore, MD, USA. [3]Department of Biomedical Engineering, Johns Hopkins University School of Medicine, Baltimore, MD, USA. [4]Translational Tissue Engineering Center, Johns Hopkins University School of Medicine, Baltimore, MD, USA. [5]Department of Chemical and Biomolecular Engineering, Johns Hopkins University, Baltimore, MD, USA. [6]Department of Materials Science and Engineering, Johns Hopkins University, Baltimore, MD, USA.

[#]These authors contributed equally to this work.

*Corresponding authors: Yizong Hu (yhu38@jhmi.edu), Tza-Huei Wang (thwang@jhu.edu), and Hai-Quan Mao (hmao@jhu.edu)

## Supplementary Discussion

The essential populational features reported in main text **Table 2**, **Fig. 3**, **Fig. 5**, and **Fig. 7** were calculated following the methods below:

### 1. Fluorescent compensation

Common for multi-color fluorescence systems, fluorescent spillovers were occasionally observed in CICS (**Supplementary Fig. 2a–c**). Compensation was therefore performed with single stained control samples. For example, we formulated TMR-PC-tagged empty LNPs with a large size and analyzed the distribution of the TMR-to-Cy5 spillover ratio across a wide range of signal intensity (**Supplementary Fig. 2f**). A fixed ratio of 0.116 was then determined as the spillover ratio. The other channels were calibrated in the same way (**Supplementary Fig. 2d, e, g**), and a compensation matrix (**Supplementary Table 2**) was calculated as the inverse of the spill-over ratio matrix with the spillover spreading matrix shown in **Supplementary Table 3**. After calibration, 93% of the spillover Cy5 signals from TMR fell below the lowest fluorescence given by individual mRNAs (*i.e.*, 80 photons in burst size, **Supplementary Fig. 2j**), and effectiveness was also confirmed in other channels (**Supplementary Fig. 2h, i**).

Through the fluorescence compensations using single stained samples, we found that only a small portion of the signals from these samples resulted in a signal intensity that exceeded the detection thresholds in the “bleeding into” channels. As shown in **Supplementary Table 4**, the percentage of the signals that bleed into the neighboring channels was only a few percent except the one from YOYO-1 to TMR. The 27.2% of the signal spillover from YOYO-1 to TMR was due to the large DNA molecules we used (Hind **III** digested lambda), which contains long strands (23 kbp) with extremely high signal intensity. This signal intensity range was not representative to the actual mRNA LNP samples as free mRNAs (996 or 1929 nt) stained by YOYO-1 would yield generally lower level of signals. For TMR single stain sample, only 2.6% of the signals caused bleeding, and the LNP formulations of interest would only have lower, and fewer such “significant signals” that needed compensation.

Therefore, the signals from LNP formulations of interest that we characterized in this paper were predominantly of a reasonably compensation-insensitive intensity. This was a result of the extensive optimizations that we performed at the beginning of this study as we carefully selected and optimized the laser sources, filters, fluorophores, and TMR-PC blending ratios to work as much as we can in a region where good signal-to-noise ratio can be maintained while minimizing the spillover ratio. This severed as the most critical factor that contributed to the accurate quantitative assessments as described in this work.

To estimate the error caused by the fluorescent compensation, we compared two of the key parameters reported by CICS (*i.e.*, mRNA payload per loaded LNP and empty LNP percentage) that were obtained with or without compensation. Here we choose two conditions (0.5% PEG lipid, and N/P = 2 at pH 7.4) as examples: 0.5% PEG lipid yielded one of the highest LNP TMR signal (**Fig. 3f**), and N/P = 2 yielded one of

the highest Cy5 signal (**Fig. 5e**) with the highest likelihood of bleeding. In addition, both conditions gave one of the highest empty LNP percentages (**Figs. 3h and 5h**). **Supplementary Table 5** shows that the compensation only minimally affected both readouts of interest.

## 2. Number-average mRNA payload

The deconvolution algorithm gives the payload capacity, (*i.e.*, number of mRNAs per LNP, $n$, and the count $w_n$). By dividing $w_n$ by the total number of LNPs, $n_{LNP}$, the weighted percentage of each LNP species encapsulating $n$ mRNAs, $w_n\%$ is obtained.

$$w_n\% = \frac{w_n}{n_{LNP}} \tag{1}$$

The number-average mRNA payload of each formulation is calculated as the sum of the multiplication of the number of mRNAs per LNP by its weighted percentage.

$$\bar{N} = \sum_{n=1}^{N} n \times w_n\% \tag{2}$$

## 3. Geometric mean of TMR and Cy5 fluorescence intensity

For each LNP formulation, the Cy5 and TMR signal of each classified species were plotted into histograms (examples: main text **Fig. 2g, h**), and the data were found to be best fitted to a log-normal distribution. Thus, the sample geometric means ($GM$) was used to describe the central tendency of the distributions, instead of arithmetic means[1], defined as:

$$GM = \exp\left(E[\ln(X)]\right) \tag{3}$$

Where $X$ is the fluorescence signal intensity as the variable, and $E[X]$ denotes the arithmetic mean of $\ln(X)$, given by:

$$E[\ln(X)] = \sum_{i=1}^{n} \frac{\ln(x_i)}{n} \tag{4}$$

For the lognormal distribution, the sample geometric mean is equivalent to the median of the distribution[2].

## 4. Calculation of the number concentrations of different species of interest

The number concentrations shown in main text **Table 2**, **Fig. 3i**, **Fig. 5j** was determined based on number-average mRNA payload and frequency of different species. At pH 4.0, the concentration of non-lipophilic complexes $n(\mathrm{TMR}^-)$ and lipophilic complexes $n(\mathrm{TMR}^+)$ can be determined by:

$$n(\mathrm{TMR}^-) \times \bar{N}(\mathrm{TMR}^-) + n(\mathrm{TMR}^+) \times \bar{N}(\mathrm{TMR}^+) = n^* \tag{5}$$

$$n(TMR^-)/n(TMR^+) = k \tag{6}$$

$n^*$ is the number concentration of all mRNA molecules in total within the formulation and is calculated based on the assumption of an average of 320 Da for each nucleotide of mRNA with the results referenced for an mRNA concentration of 20 µg/mL; $\bar{N}$ is number-average mRNA payload of the species determined by CICS; and $k$ is frequency ratio between the two species determined by CICS. The concentration of empty LNPs was then calculated by the ratio of empty LNPs $(\mathrm{TMR^+|Cy5^-})$ to lipophilic complexes $(\mathrm{TMR^+|Cy5^+})$ in all TMR-positive events given by CICS.

At pH 7.4, the concentration of mRNA-loaded particles $n(\mathrm{TMR^+|YOYO^-|Cy5^+})$ is determined by:

$$n(TMR^+|YOYO^-|Cy5^+) \times \bar{N}(TMR^+|YOYO^-|Cy5^+) = n^* \times EE\% \tag{7}$$

$\mathrm{EE\%}$ is the encapsulation efficiency determined by RiboGreen assay (see Methods), and $\bar{N}$ was given by CICS assessments of the samples.

## 5. Encapsulation efficiency (EE%) characterized by CICS.

After characterization of the payload distribution and capacity by CICS, the EE% can be given by:

$$\mathrm{EE\%_{CICS}} = \frac{n(\mathrm{TMR^+|YOYO^-|Cy5^+}) \times \bar{N}(\mathrm{TMR^+|YOYO^-|Cy5^+})}{n(\mathrm{TMR^+|YOYO^-|Cy5^+}) \times \bar{N}(\mathrm{TMR^+|YOYO^-|Cy5^+}) + n(\mathrm{TMR^{+/-}|YOYO^+|Cy5^+}) \times \bar{N}(\mathrm{TMR^{+/-}|YOYO^+|Cy5^+})} \tag{8}$$

For most of the formulations, CICS showed only slight differences compared with RiboGreen (**Supplementary Figure 4**). We attribute these differences to intrinsic deviations of both methods. RiboGreen is a colorimetrical method based on comparison of the binding degree of the dye to free mRNA molecules vs. to all mRNA molecules released by LNP disruption. The biggest origin of deviation is uncertainty of dye penetration into LNPs and dye binding to lipid components that will trigger fluorescence emissions, which cannot be calibrated by standard curves. Our CICS method used fluorescence coincidence analysis to identify YOYO-1$^-$, TMR$^+$ and Cy5$^+$ events first (main text **Table 1**), and then use the absolute mRNA amount quantified in these mRNA-loaded LNPs to calculate EE% through dividing it by the total mRNA fluorescence quantified (described in Supporting Discussion). The biggest origin of deviation we anticipate is a certain degree of uncertainty of YOYO-1 penetration and TMR thresholding level to define TMR$^+$.

The only outlier was the formulation of N/P = 1, for which CICS and RiboGreen showed drastically different results. This formulation is very special because it is anticipated that the mRNA encapsulation would be poor as the ionizable lipids were insufficient to neutralize all the negative charges on mRNA molecules. We hypothesize that there would still be certain degrees of lipid complexation onto the mRNA molecules, however, the complexation could not fully protect the mRNAs from binding to RiboGreen dye. RiboGreen therefore gave a very low EE% reading of 27.8%. However, the same degree of lipid complexation might accommodate enough TMR-PC lipids to make its signal exceed the detection threshold; besides, the

YOYO-1 dye we used might not penetrate to bind to mRNAs as well as RiboGreen. We found that in this case most of the Cy5 events were still recognized as mRNA-loaded LNPs by CICS and it gave a high EE% reading of 82.9%. This difference should not be recognized as an error, but it represents different aspects of the encapsulation of mRNA.

## Supplementary Tables

**Supplementary Table 1**. Comparison of LNPs formulated with or without fluorescent labels at pH 7.4

| | Z-average diameter (nm) | Zeta-potential (mV) | Encapsulation efficiency |
|---|---|---|---|
| LNPs formulated with non-labeled mRNA and no labeled lipid | 113.0 ± 1.4 | -3.2 ± 1.4 | 95.6% ± 0.6% by RiboGreen assay |
| LNPs formulated by Cy5-mRNA with TMR-lipid added | 120.5 ± 6.0* | -6.3 ± 1.3* | 94.2% ± 3.6%* by RiboGreen assay |

* These data points were reported in **Table 2** in the main text of the manuscript.

**Implications:**

The zeta-potential (by phase analysis light scattering) values agree with literature reports of this LNP formulation, which were usually described as "neutral"[3] or (slightly) negatively charged[4], with exact values ranging from +0.5 ~ +0.9 mV[5], -2.91 mV[6], around -5 mV[7], to around -8 mV[4]. The slight differences seen in the z-average and zeta-potential between the two groups were non-substantial, according to guidelines on interpretation of dynamic light scattering and phase analysis light scattering data[8].

**Supplementary Table 2**. Compensation matrix used in quantitative assessments

| | YOYO-1 | TMR | Cy5 |
|---|---|---|---|
| YOYO-1 | 1 | -0.071 | 0 |
| TMR | -0.033 | 1 | -0.116 |
| Cy5 | 0 | -0.007 | 1 |

**Supplementary Table 3**. Spillover spreading matrix (SSM) of the 3-color CICS experiments

| | | Detector (Filter wavelength center/bandwidth in nm) | | |
|---|---|---|---|---|
| | | 520/28 | 575/25 | 676/37 |
| Fluorophore | YOYO-1 | N/A | 0.7909 | 0 |
| | TMR | 0.648 | N/A | 1.0132 |
| | Cy5 | 0 | 0.9368 | N/A |

The spillover spreading matrix (SSM) was determined following the equations in a previous report[9]. Taking the YOYO-1 fluorescence spillover into 575/25 nm APD detector as an example, the spillover spread value SS is given by:

$$SS_{575/25}^{YOYO-1} = \frac{\Delta\sigma_{575/25}}{\sqrt{\Delta F_{YOYO-1}}} \tag{9}$$

Where $\Delta\sigma_{575/25}$ is the incremental standard deviation arising from the spectral overlap and $\Delta F_{YOYO-1}$ is the difference in median fluorescence of the non-stained reference and YOYO-1 single-stained sample in the primary parameter (YOYO-1 on the 520/28-nm detector). The off-diagonal elements are the intrinsic spillover spreading value and the low values indicate that the spillover spreading has minimal effect on the sensitivity and quantification of the 3-color fluorescence signals.

**Supplementary Table 4**. The frequencies of bleed-through events using single stained samples

| | | % Signal bleeding into | | |
|---|---|---|---|---|
| | | YOYO-1 | TMR | Cy5 |
| From | DNA-YOYO-1 | N/A | 27.2% | 0.2% |
| | mRNA LNP, 0.25% PEG TMR only | 1.2% | N/A | 1.5% |
| | mRNA LNP, 0.25% PEG Cy5 only | 1.2% | 2.6% | N/A |

**Supplementary Table 5**. Comparison of the empty LNP percentage and average mRNA per loaded LNP with or without fluorescence compensation for two representative LNP formulations

| | | **Empty LNP%** | | **Average mRNA copy per loaded LNP** | |
|---|---|---|---|---|---|
| Condition | Replicate | w/ compensation | w/o compensation | w/ compensation | w/o compensation |
| 0.5% PEG lipid | #1 | 76.9% | 76.8% | 5.19 | 5.13 |
| | #2 | 85.3% | 85.2% | 7.30 | 7.31 |
| | #3 | 76.3% | 76.2% | 8.18 | 8.12 |
| N/P = 2 | #1 | 82.4% | 82.4% | 6.86 | 6.78 |
| | #2 | 83.7% | 83.7% | 7.36 | 7.36 |
| | #3 | 71.9% | 71.9% | 11.01 | 10.95 |

## Supplementary Methods

**Airyscan super-resolution imaging of mRNA LNPs**. The benchmark mRNA LNP formulation at pH = 7.4 was diluted to a concentration of 2 µg mRNA/mL, and 5 µL was dropped on a cover glass (#1.5 thickness, Electron Microscopy Services, Cat# 72230). A glycerol-based resin (ProLong Diamond Antifade Mountant from Invitrogen, Cat# P36961) was heated to 37°C to fully liquefy with reduced viscosity, and a 20 µL drop was applied on top of a glass slide. The cover glass with the LNP drop was then flipped and glued onto the glass slide upon contact with the resin. This step mixed the LNP suspension with the liquid resin. As temperature dropped, viscosity increased significantly and thus embedded the LNPs. The sample was subsequently dried under room temperature for an additional 48 hours to fully solidify the resin. The sample was then assessed using a LSM800 confocal microscope (Zeiss) equipped with the Airyscan super-resolution modality.

## Supplementary Figures

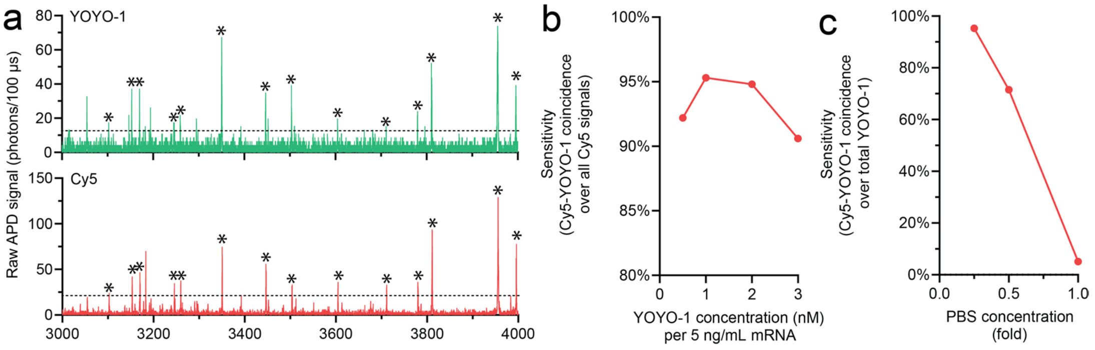


**Supplementary Figure 1. Optimization of YOYO-1 staining conditions for CICS detection of free mRNA with highest sensitivity. (a)** YOYO-1 stains free mRNAs in solution and was recognized by CICS. **(b)** Optimization of the ratio between YOYO-1 and mRNA, justifying a final selection of 1 nM YOYO-1 per 5 ng/mL mRNA. **(c)** Optimization of the ionic strength for YOYO-1 staining of free mRNA, justifying a final selection of 0.25× PBS. With the optimal conditions used, the sensitivity for detection of free mRNA reached 95%.

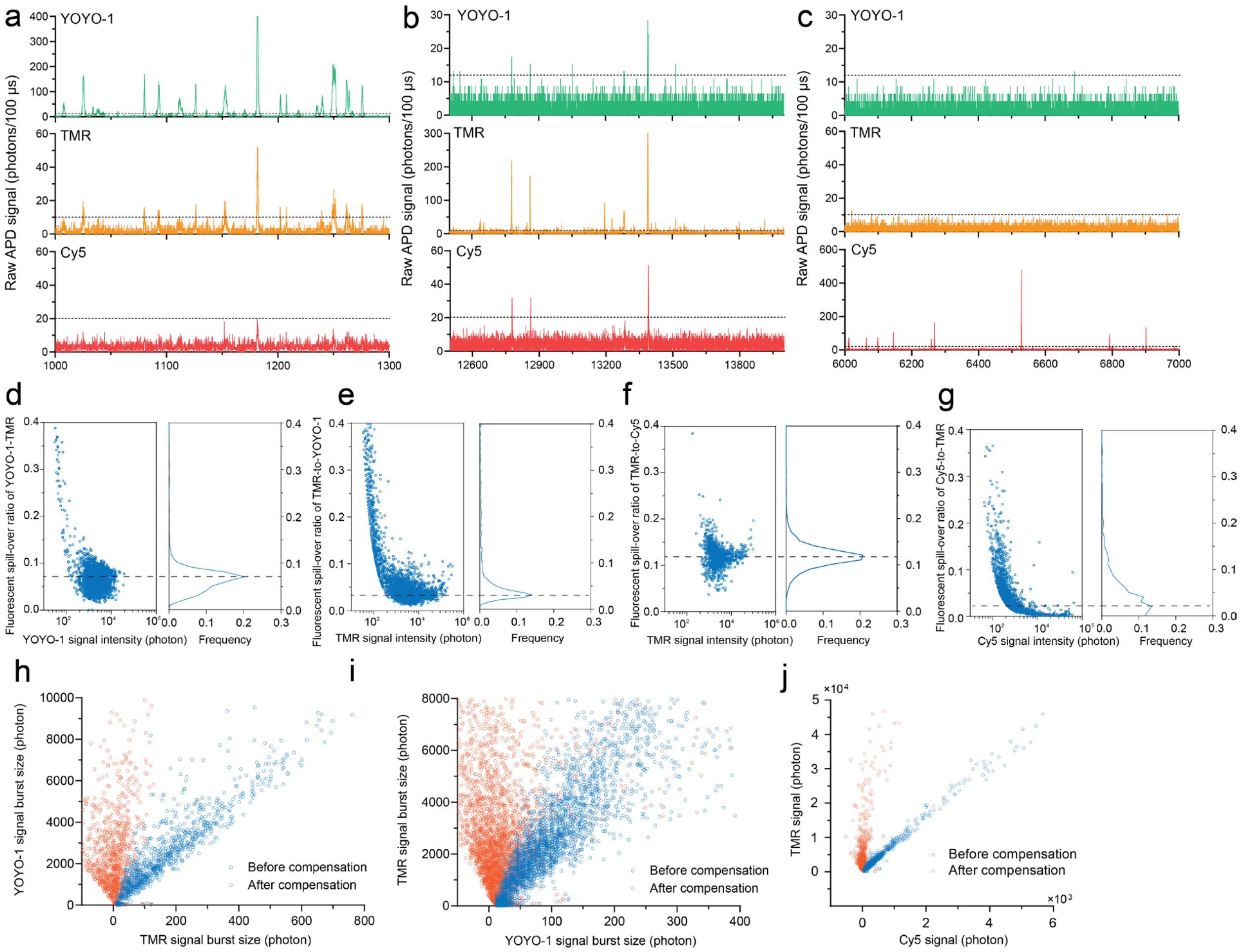


**Supplementary Figure 2. Determination of the compensation matrix used throughout the study. (a–c)** The spill-over of **(a)** YOYO-1; **(b)** TMR; and **(c)** Cy5 signals into the adjacent channels. In **(a)**, YOYO-1 signals have frequent spill-overs into the TMR channel and negligible spill-over into the Cy5 channel; In **(b)**, TMR signals have frequent spill-overs into both the YOYO-1 and Cy5 channels; In **(c)**, Cy5 signals have occasional spill-over into the TMR channel, with even rarer spill-over into the YOYO-1 channel. **(d–g)** The ratio of spill-over signal intensity to the control fluorescence signal intensity in the original channel and its distribution of all spill-over events, in which the mode of the distribution was determined as the spill-over ratio. After application of the compensation matrix (**Supplementary Table 2**), the spill-overs of **(d)** YOYO-1 signals into TMR channel; **(e)** TMR signals into YOYO-1 channel; **(f)** TMR signals into Cy5 channel; and **(g)** Cy5 signals into TMR channel were corrected. Application of compensation successfully corrected most of the spill-over signals **(h)** from YOYO-1 channel to TMR channel; **(i)** from TMR channel to YOYO-1 channel; as well as **(j)** from TMR channel to Cy5 channel.

Each control was chosen with the purpose of maximizing the range of signal intensity for a most representative compensation factor. For YOYO-1 channel, the control sample was Hind III digested lambda

DNA (New England Biolabs, Cat# B7025) stained by YOYO-1; For TMR channel, the control sample was the formulation with 0.25% PEG with TMR-PC being the only fluorescent component (*i.e.*, with non-fluorescent luciferase mRNA); For Cy5 channel the control sample was the same 0.25% PEG formulation with Cy5-mRNA but without addition of TMR-PC. Each control sample was tested at the same experimental conditions as the actual samples and went through the single fluorescence analysis. The spill-over signal from a given channel to the other two channels were identified by coincidence analysis, and the ratio of the spill-over signal intensity to the control fluorescence intensity in the original channel was calculated. The spill-over ratio was determined as the mode of the spillover distribution (dashed lines in **Supplementary Fig. 2d–g**). A fluorescent spillover matrix was obtained with all the spill-over ratios determined. The fluorescent compensation matrix was calculated as the inverse of the spill-over matrix (**Supplementary Table 2**). The performance of the fluorescence compensation was evaluated by the percentage of the spill-over signal after correction that fell below the minimal value of the signal in that channel, with the account of the percentage of the spill-over rate.

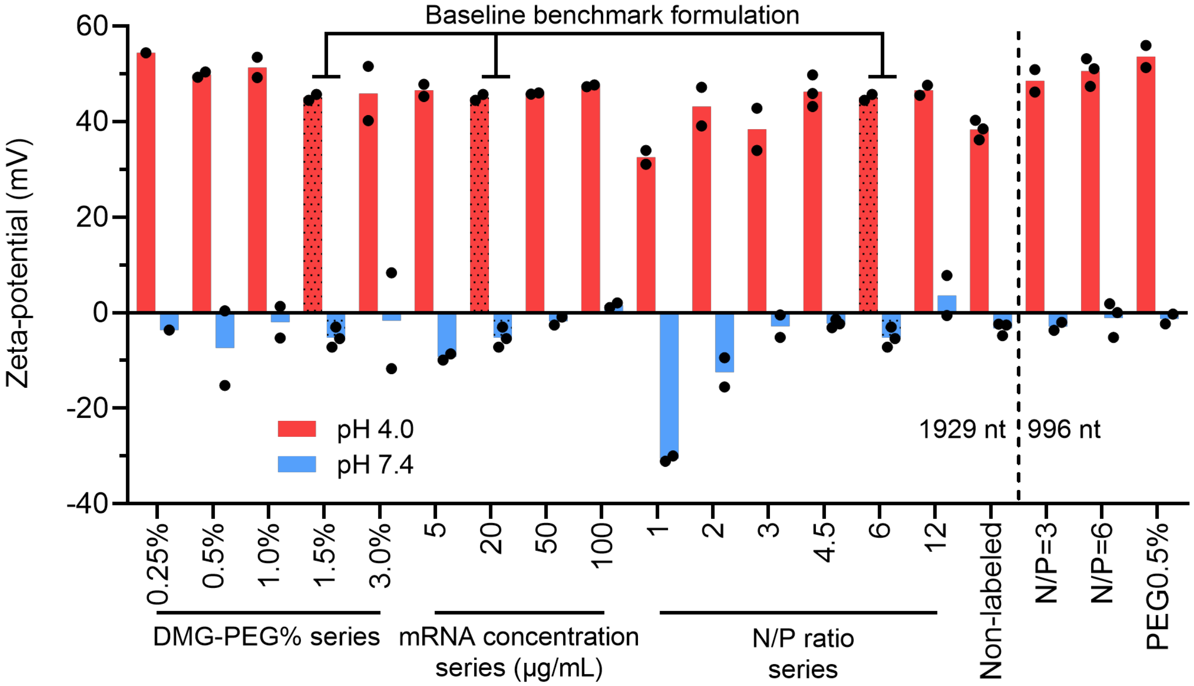


**Supplementary Figure 3. Surface charge (zeta-potential) of all formulations tested in this study.** The ionizable lipid used in this study, *i.e.*, DLin-MC3-DMA, has an apparent pKa value of 6.44. At pH 4.0, almost 100% of the lipids were positively charged, rendering a highly positive zeta-potential to the LNPs; At pH 7.4, around 90% of the lipids lose the positive charge. At an N/P ratio ≥ 3, this resulted in a near-neutral or slightly negative zeta-potential of the LNPs. It is noticeable that N/P = 1 at pH 4.0 did not yield charge-neutral complexes, presumably due to reduced negative charges on mRNA molecules due to the covalently attached Cy5. The "Non-labeled" group was formulated with the same recipe of the baseline benchmark formulation, but the mRNA used was 1929-nt luciferase mRNA without Cy5 and no fluorescent TMR-PC helper lipid was added. The data showed that the presence of Cy5 on the mRNA and TMR on the helper lipid did not significantly alter the charge property at either pH 4.0 or pH 7.4, nor the surface charge transition from pH 4.0 to pH 7.4. Each data point shown in the figure represents measurement result from an independent experiment (formulation of mRNA LNPs from raw materials and then carry out measurement).

The bars presented in this figure represent the mean value out of data points collected from independent experiments (formulating LNPs from raw materials and then applying analysis).

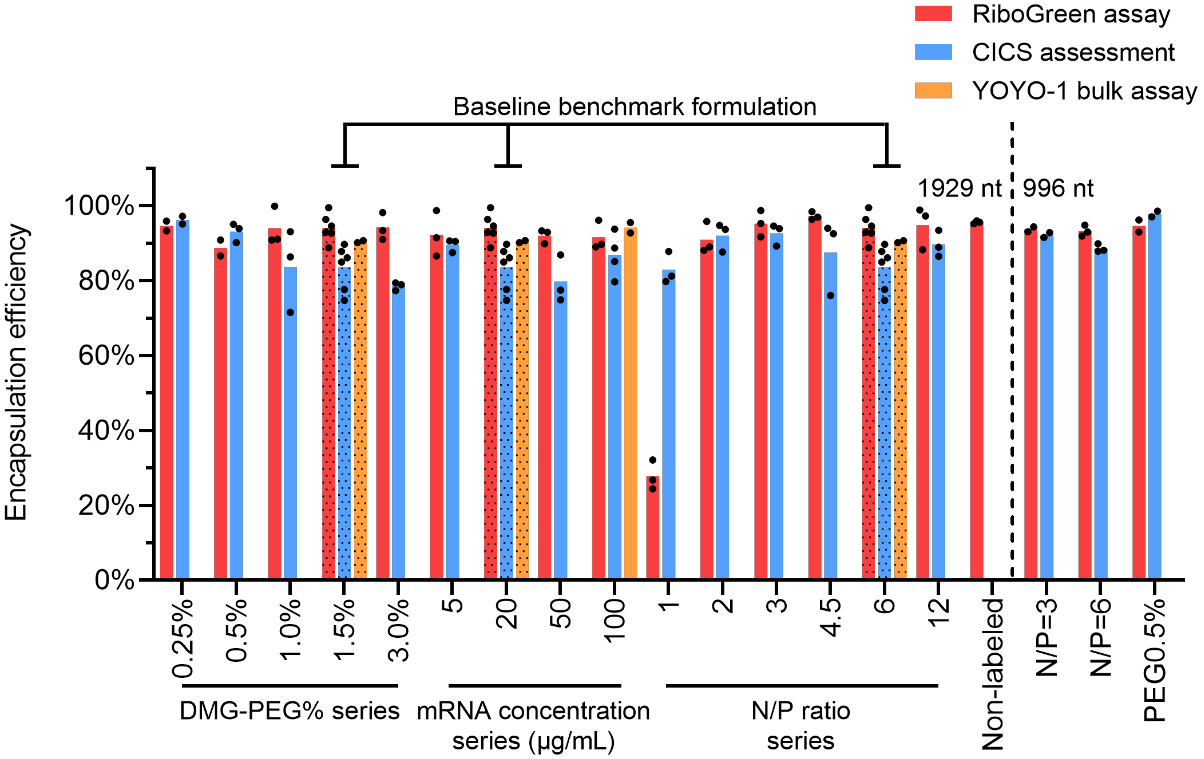


**Supplementary Figure 4. Encapsulation efficiency of all formulations tested in this study characterized by RiboGreen assay, YOYO-1 assay or CICS.** The assessment of encapsulation efficiency using CICS was carried out by obtaining the ratio of total mRNA copies from identified free mRNA events to total mRNA copies from identified mRNA-loaded LNP events. Each data point shown in the figure represents measurement result from an independent experiment (formulation of mRNA LNPs from raw materials and then carry out measurement).

The "Non-labeled" group was formulated with the same recipe of the baseline benchmark formulation, but the mRNA used was 1929-nt luciferase mRNA without Cy5 and no fluorescent TMR-PC helper lipid was added. The data showed that Cy5-mRNA was encapsulated into LNPs equally well as non-labeled mRNAs, and addition of TMR-PC helper lipid did not negatively influence the encapsulation. Also, the non-labeled mRNA LNPs held a z-average diameter of 85.7 ± 3.3 nm at pH 4.0, and of 113 ± 1.4 nm at pH 7.4, which were similar to those of LNPs formulated with Cy5-mRNA and TMR-PC (Table 2 in the main text). We therefore concluded that the presence of fluorescent tags on mRNA and the helper lipid did not influence the assembly of the mRNA LNPs, so the results presented in this paper are representative.

The bars presented in this figure represent the mean value out of data points collected from independent experiments (formulating LNPs from raw materials and then applying analysis).

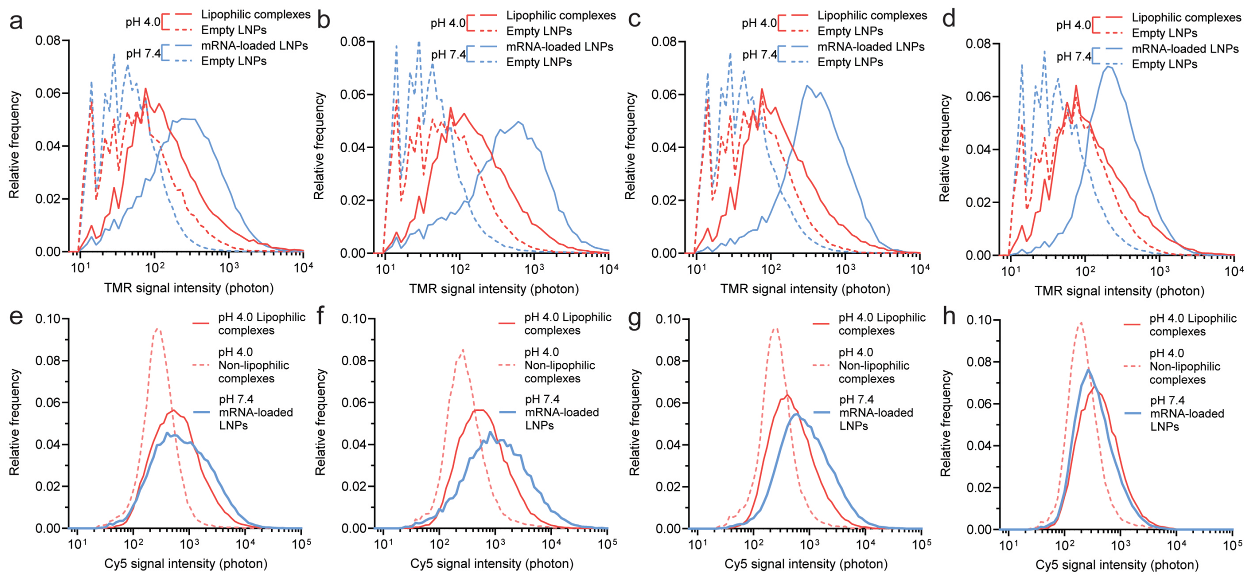


**Supplementary Figure 5. Signal intensity histograms of the LNP formulations with varied concentration of the PEG lipid. (a–d)** The TMR signal intensity histograms of different species at pH 4.0 or 7.4 for the formulation with a molar dosage of PEG lipid of **(a)** 0.25%; **(b)** 0.5%; **(c)** 1.0%; and **(d)** 3.0%. Note that the histograms for the formulation with PEG% = 1.5% is shown in main text **Fig. 2g**. **(e–h)** The Cy5 signal intensity histograms of different species at pH 4.0 or 7.4 for the formulation with a molar dosage of PEG lipid of **(e)** 0.25%; **(f)** 0.5%; **(g)** 1.0%; and **(h)** 3.0%. These Cy5 histograms were used in the deconvolution algorithm to resolve the mRNA payload in LNPs. Note that the histograms for the formulation with PEG% = 1.5% is shown in main text **Fig. 2h**.

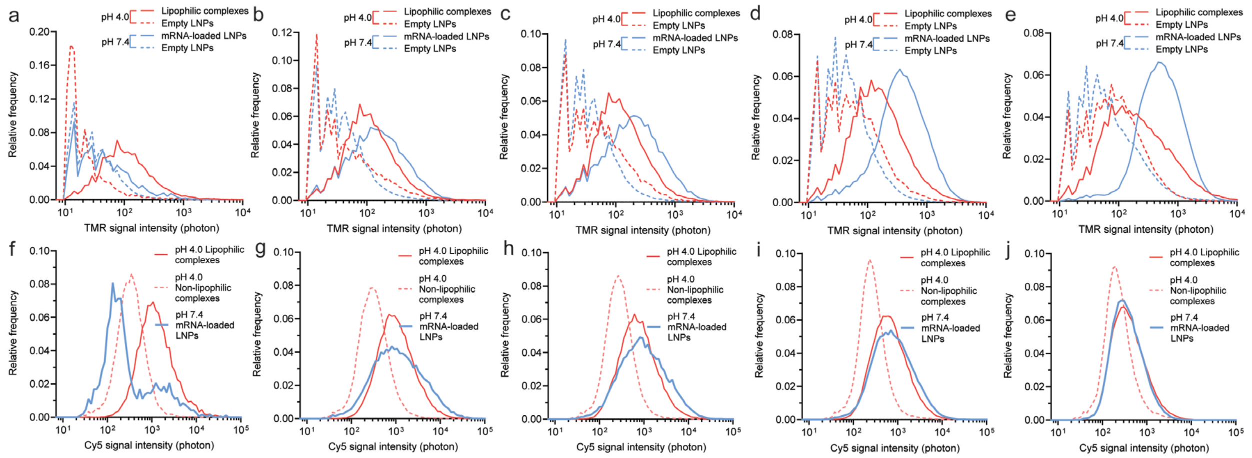


**Supplementary Figure 6. Signal intensity histograms of the LNP formulations with varied N/P ratio.** **(a–e)** The TMR signal intensity histograms of different species at pH 4.0 or 7.4 for the formulation with N/P ratio of **(a)** 1; **(b)** 2; **(c)** 3; **(d)** 4.5; and **(e)** 12. Note that the histograms for the formulation with N/P = 6 is shown in main text **Fig. 2g**. **(f–j)** The Cy5 signal intensity histograms of different species at pH 4.0 or 7.4 for the formulation with N/P ratio of **(f)** 1; **(g)** 2; **(h)** 3; **(i)** 4.5; and **(j)** 12. These Cy5 histograms were used in the deconvolution algorithm to resolve the mRNA payload in LNPs. Note that the histograms for the formulation with N/P = 6 is shown in main text **Fig. 2h**.

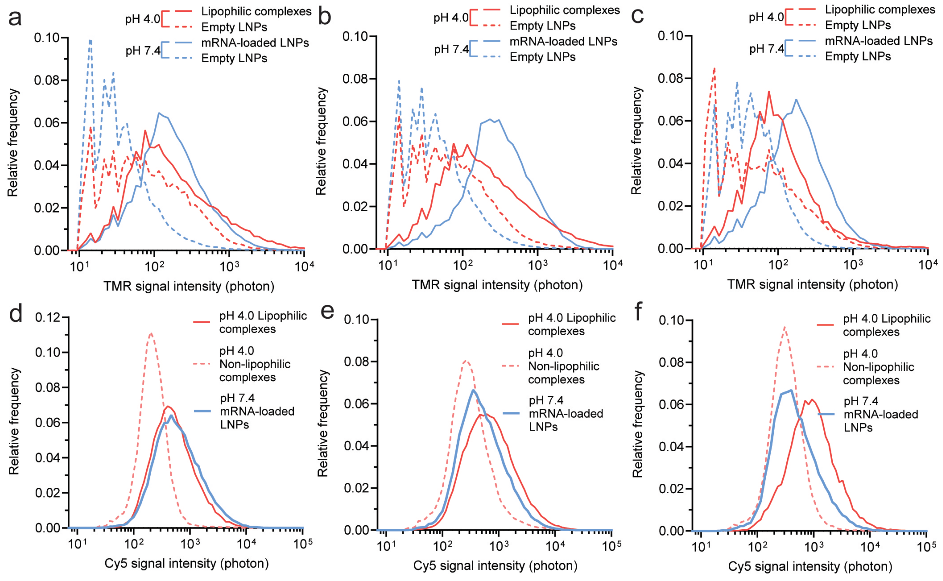


**Supplementary Figure 7. Signal intensity histograms of the LNP formulations with varied mRNA concentrations. (a–c)** The TMR signal intensity histograms of different species at pH 4.0 or 7.4 for the formulation at an mRNA concentration of **(a)** 5; **(b)** 50; and **(c)** 100 µg/mL. Note that the histograms for the formulation with 20 µg/mL is shown in main text **Fig. 2g**. **(d–f)** The Cy5 signal intensity histograms of different species at pH 4.0 or 7.4 for the formulation at an mRNA concentration of **(d)** 5; **(e)** 50; and **(f)** 100 µg/mL; These Cy5 histograms were used in the deconvolution algorithm to resolve the mRNA payload in LNPs. Note that the histograms for the formulation with 20 µg/mL is shown in main text **Fig. 2h**.

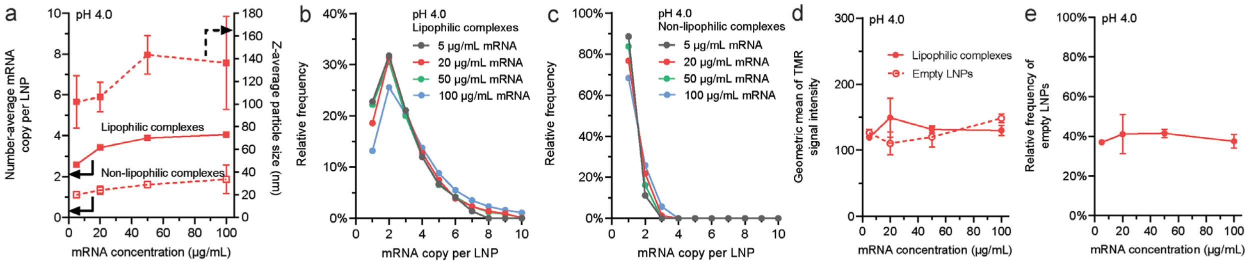


**Supplementary Figure 8. mRNA payload characteristics for LNPs at different mRNA concentrations at pH 4.0.** The effect of mRNA (and lipids) concentration on **(a)** the z-average particle size and number-average mRNA payload; **(b)** the payload distribution of lipophilic complexes; **(c)** the payload distribution of non-lipophilic complexes; **(d)** the relative helper lipid content in lipophilic complexes or empty LNPs, as well as **(e)** the fraction of empty LNPs at pH 4.0. In (a), (c) and (e), data are represented as mean value +/- SD, derived from n = 3 independent experiments (formulating LNPs from raw materials and then applying CICS analysis), except for 20 µg/mL where n = 6.

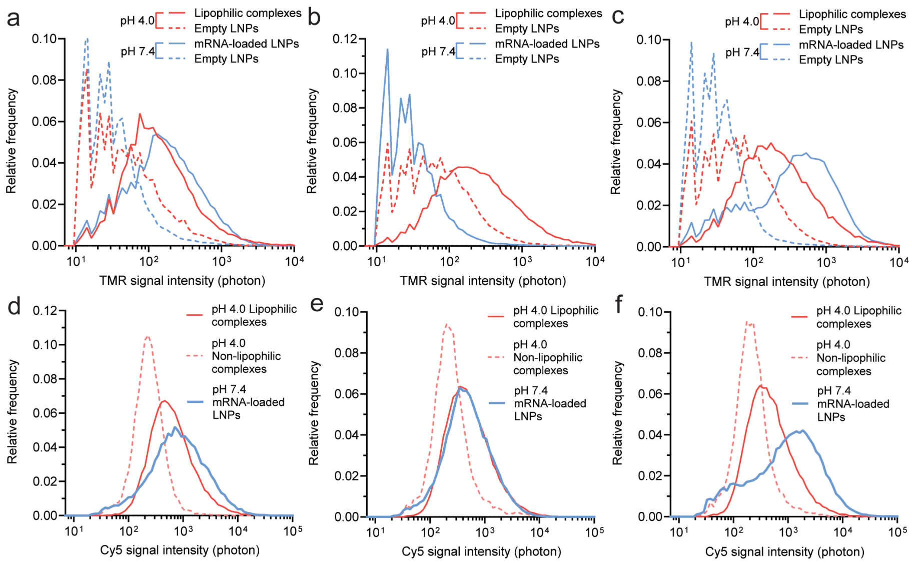


**Supplementary Figure 9. Signal intensity histograms of the LNP formulations prepared by an mRNA with a halved (996 nt) size. (a–c)** The TMR signal intensity histograms of different species at pH 4.0 or 7.4 for the formulation with **(a)** N/P = 3; **(b)** N/P = 6; and **(c)** 0.5% PEG lipid. **(d–f)** The Cy5 signal intensity histograms of different species at pH 4.0 or 7.4 for the formulation with **(d)** N/P = 3; **(e)** N/P = 6; **(f)** 0.5% PEG lipid; These Cy5 histograms were used in the deconvolution algorithm to resolve the mRNA payload in LNPs.

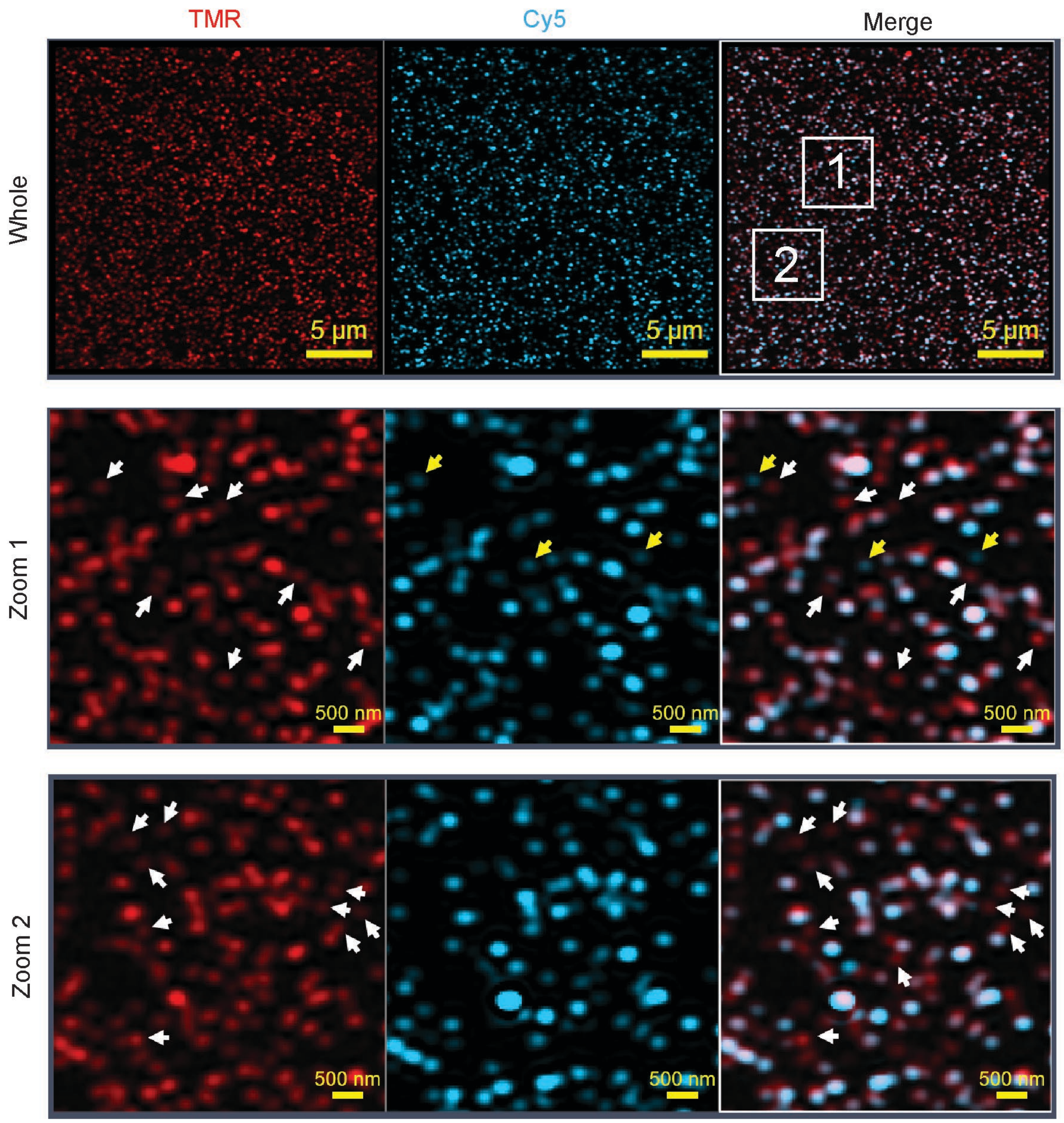


**Supplementary Figure 10. Airyscan super-resolution imaging of embedded benchmark mRNA LNP formulation at pH 7.4.** The lane “Whole” shows a full imaging area, while “Zoom 1” and “Zoom 2” show the zoom-in images of the areas labeled in “Whole”. The white arrows in the zoom-in area 1 or 2 showcase presence of a large quantity of empty LNPs ($TMR^+$ $Cy5^-$), while the yellow arrows in the zoom-in area 1 showcase presence of unencapsulated mRNAs ($TMR^-$ $Cy5^+$). The images shown are representative images from 2 independent sample preparations and 20 fields examined for each preparation, for which the findings were consistent.

**Implications:** These images qualitatively confirmed the presence of empty LNPs in the formulation. However, we think there is a possibility that these fluorescence images underestimated the ratio of empty LNPs because CICS revealed that the average TMR signal intensity of mRNA-loaded LNPs was nearly one magnitude higher than that of empty LNPs (main text **Fig. 2g**).

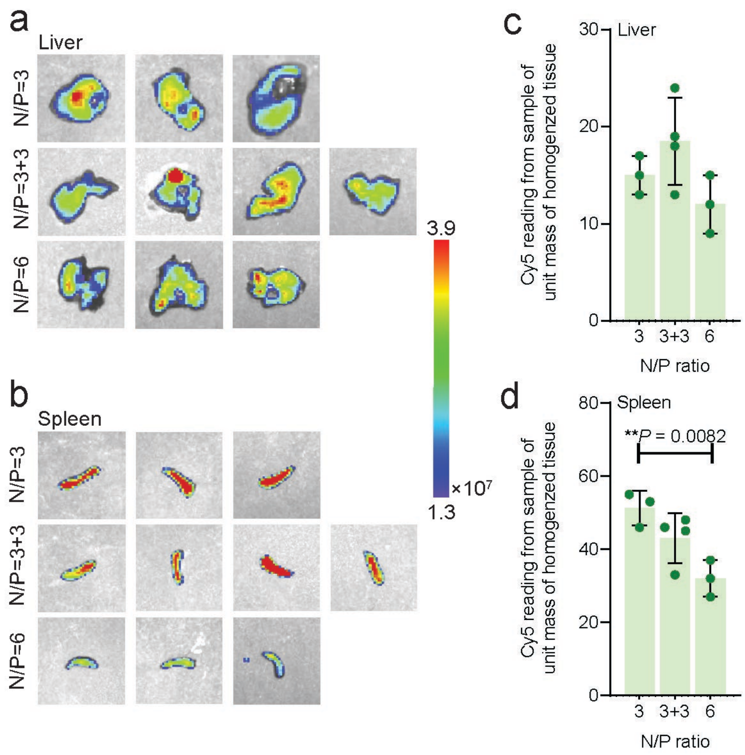


**Supplementary Figure 11. Biodistribution of mRNA LNP formulations tested *in vivo*.** In this experiment, the same animal experimental procedures as described in the **Methods** section in the main text were used with several modifications: (1) The cargo was Cy5-mRNA for the purpose of tracking the biodistribution of the mRNA-loaded LNPs; (2) The IVIS live-animal imaging was carried out to harvested organs at 4 hours post-injection with fluorescence detection mode to Cy5; (3) The homogenized organ solution samples were analyzed by a plate reader in detection of Cy5. The results are shown in **(a)** and **(c)** for liver, and in **(b)** and **(d)** for spleen. For statistically analysis in **(d)**, an unpaired t test was performed with ** denoting $p$ (two sided) < 0.01. In **(a)** and **(b)**, the scale represents fluorescence radiant efficiency with the unit of (p/sec/cm$^2$/sr)/(μW/cm$^2$). In **(c)** and **(d)**, n = 3 for N/P = 3 and N/P = 6 groups, n = 4 for N/P = 3+3 group, and the data are presented as mean value +/- SD.

The results show that the biodistribution of the mRNA LNPs to the liver did not differ significantly across different groups, meaning that the decrease of transfection efficiency in the liver caused by addition of extra empty LNPs into the N/P = 3 formulation could not be explained by different biodistribution profiles. The biodistribution to spleen was significantly lower for N/P = 6 compared to the other two formulations based on N/P = 3, and it correlates with the lower transfection efficiency of N/P = 6 formulation in the spleen. However, we could not conclude that the biodistribution profile was the only reason that led to the transfection profiles.

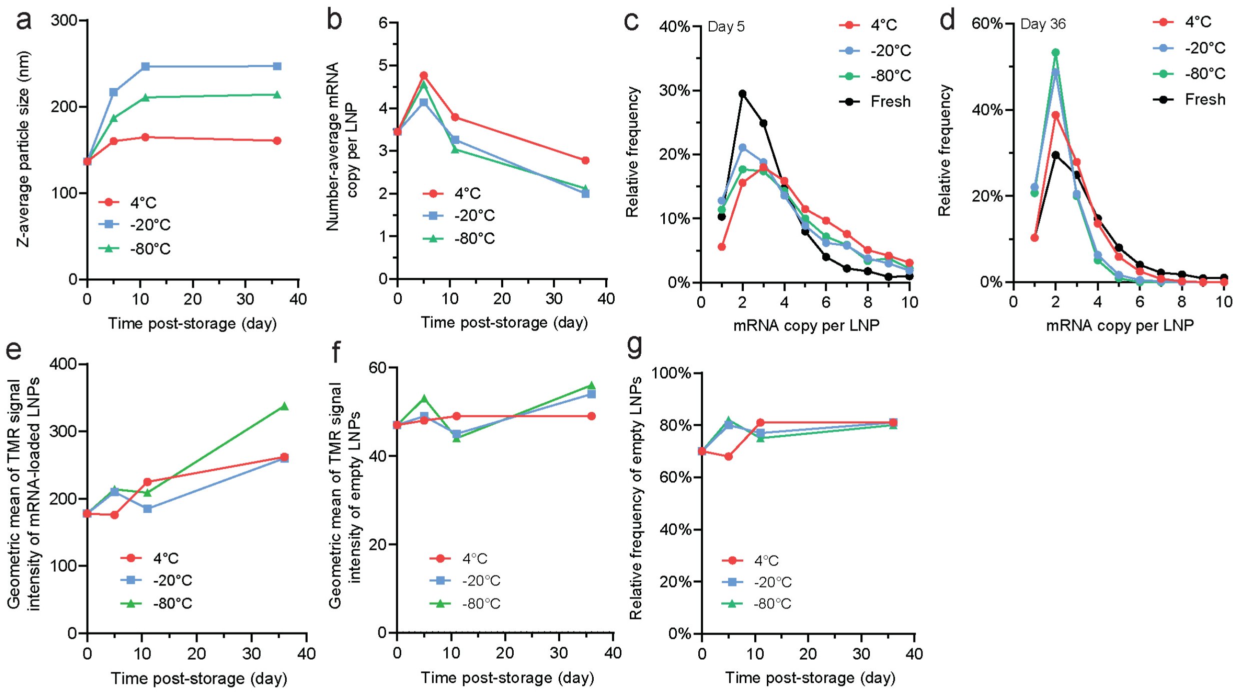


**Supplementary Figure 12. Monitoring payload distribution and capacity along with the storage of mRNA LNPs under different conditions.** In this experiment, the LNPs were formulated with 5% sucrose as a validated single cryo-protectant. Upon formulation to pH 7.4, the samples were stored either in 4°C, -20°C, or -80°C, which are common storage conditions for mRNA LNPs. At 5 days, 11 days and 36 days post-storage, the samples were warmed up to room temperature and analyzed by CICS. The **(a)** z-average size. **(b)** average mRNA copy per nanoparticle among mRNA-loaded LNPs; **(e)** geometric mean of TMR signals (indicator of relative helper lipid content) of mRNA-loaded LNPs, **(f)** geometric mean of TMR signals of empty LNPs, and **(g)** fraction of empty LNPs are shown. The payload distribution profiles of the samples assessed at **(c)** 5 days, and **(d)** 36 days post-storage, respectively, are also shown to illustrate the payload distribution changes. Throughout this figure, data are presented from the measurement result of a single experiment (n = 1).

While we were unable to perform this experiment with the formulation as in the real mRNA LNP product on the market, an existing publication has validated 5% sucrose as an effective single cryo-protectant for long-term low-temperature storage[10]. Under this storage condition, a series of changes in the payload distribution and capacity of the mRNA LNP formulation was observed. Firstly, no significant differences were observed between the samples stored under -20°C and those under -80°C (**Supplementary Figure 12b–g**) except -20°C resulted in greater size increase upon thawing (**Supplementary Figure 12a**). The samples stored under 4°C did not show appreciable quality drop over the entire storage period of 36 days. While numerous reports (including the paper we reference to[10]) clearly demonstrated that the biological activity of mRNA LNP formulations quickly decreased to zero a few days upon stored under 4°C, we showed through our experiments that it was not due to collapse of the LNP structure or assembly features. This agrees with

literature report that hydrolysis of the mRNA molecules might be the major culprit for instability of mRNA LNP formulations under elevated temperature[11]. Even after mRNA molecules are broken down to shorter, unfunctional strands, they are still complexed in the LNP core, leaving payload distribution and capacity of the LNPs unchanged. We also observed a trend of increasing helper lipid content in the mRNA-loaded LNPs, but it remains unclear to us about the underlying mechanisms.

**Supplementary References**


1. Feng, C., Wang, H. & Tu, X.M. Geometric Mean of Nonnegative Random Variable. *Communications in Statistics - Theory and Methods* **42**, 2714-2717 (2013).
2. Vogel, R.M. The geometric mean? *Communications in Statistics - Theory and Methods*, 1-13 (2020).
3. Semple, S.C. et al. Rational design of cationic lipids for siRNA delivery. *Nature Biotechnology* **28**, 172-176 (2010).
4. Carrasco, M.J. et al. Ionization and structural properties of mRNA lipid nanoparticles influence expression in intramuscular and intravascular administration. *Communications Biology* **4**, 956 (2021).
5. Veiga, N. et al. Cell specific delivery of modified mRNA expressing therapeutic proteins to leukocytes. *Nature Communications* **9**, 4493 (2018).
6. Ball, R.L., Hajj, K.A., Vizelman, J., Bajaj, P. & Whitehead, K.A. Lipid Nanoparticle Formulations for Enhanced Co-delivery of siRNA and mRNA. *Nano Letters* **18**, 3814-3822 (2018).
7. Larson, N.R. et al. pH-Dependent Phase Behavior and Stability of Cationic Lipid–mRNA Nanoparticles. *Journal of Pharmaceutical Sciences* **111**, 690-698 (2022).
8. Bhattacharjee, S. DLS and zeta potential – What they are and what they are not? *Journal of Controlled Release* **235**, 337-351 (2016).
9. Nguyen, R., Perfetto, S., Mahnke, Y.D., Chattopadhyay, P. & Roederer, M. Quantifying spillover spreading for comparing instrument performance and aiding in multicolor panel design. *Cytometry Part A* **83A**, 306-315 (2013).
10. Zhao, P. et al. Long-term storage of lipid-like nanoparticles for mRNA delivery. *Bioactive Materials* **5**, 358-363 (2020).
11. Schoenmaker, L. et al. mRNA-lipid nanoparticle COVID-19 vaccines: Structure and stability. *International Journal of Pharmaceutics* **601**, 120586 (2021).
