# Supplementary material for: Payload distribution and capacity of mRNA lipid nanoparticles
Source: Nat Commun. 2022 Sep 23;13:5561. doi: 10.1038/s41467-022-33157-4 (PMC9508184; doi:10.1038/s41467-022-33157-4)
Supplement: Supplementary file 3 — Reporting Summary [file 41467_2022_33157_MOESM3_ESM.pdf]

nature portfolio

Corresponding author(s): Yizong Hu, Tza-Huei Wang, Hai-Quan Mao

Last updated by author(s): Aug 13, 2022

Reporting Summary

Nature Portfolio wishes to improve the reproducibility of the work that we publish. This form provides structure for consistency and transparency in reporting. For further information on Nature Portfolio policies, see our Editorial Policies and the Editorial Policy Checklist.

Statistics

For all statistical analyses, confirm that the following items are present in the figure legend, table legend, main text, or Methods section.

n/a	Confirmed	
☐	☒	The exact sample size (*n*) for each experimental group/condition, given as a discrete number and unit of measurement
☐	☒	A statement on whether measurements were taken from distinct samples or whether the same sample was measured repeatedly
☐	☒	The statistical test(s) used AND whether they are one- or two-sided<br>*Only common tests should be described solely by name; describe more complex techniques in the Methods section.*
☒	☐	A description of all covariates tested
☒	☐	A description of any assumptions or corrections, such as tests of normality and adjustment for multiple comparisons
☐	☒	A full description of the statistical parameters including central tendency (e.g. means) or other basic estimates (e.g. regression coefficient) AND variation (e.g. standard deviation) or associated estimates of uncertainty (e.g. confidence intervals)
☐	☒	For null hypothesis testing, the test statistic (e.g. *F*, *t*, *r*) with confidence intervals, effect sizes, degrees of freedom and *P* value noted<br>*Give P values as exact values whenever suitable.*
☒	☐	For Bayesian analysis, information on the choice of priors and Markov chain Monte Carlo settings
☒	☐	For hierarchical and complex designs, identification of the appropriate level for tests and full reporting of outcomes
☒	☐	Estimates of effect sizes (e.g. Cohen's *d*, Pearson's *r*), indicating how they were calculated

Our web collection on statistics for biologists contains articles on many of the points above.

Software and code

Policy information about availability of computer code

Data collection	LabVIEW (Version 2020, National Instruments) was used for CICS data collection.
Data analysis	Prism (Version 9, GraphPad) was used for data visualization and statistical analysis. MATLAB (Version 2021a, MathWorks) was used for data analysis.

For manuscripts utilizing custom algorithms or software that are central to the research but not yet described in published literature, software must be made available to editors and reviewers. We strongly encourage code deposition in a community repository (e.g. GitHub). See the Nature Portfolio guidelines for submitting code & software for further information.

Data

Policy information about availability of data

All manuscripts must include a data availability statement. This statement should provide the following information, where applicable:

- Accession codes, unique identifiers, or web links for publicly available datasets
- A description of any restrictions on data availability
- For clinical datasets or third party data, please ensure that the statement adheres to our policy

All data needed to evaluate the conclusions in this paper are present in the paper and / or the Supplementary Materials. Source data are provided with this paper.

Field-specific reporting

Please select the one below that is the best fit for your research. If you are not sure, read the appropriate sections before making your selection.

- [x] Life sciences
- [ ] Behavioural & social sciences
- [ ] Ecological, evolutionary & environmental sciences

For a reference copy of the document with all sections, see nature.com/documents/nr-reporting-summary-flat.pdf

Life sciences study design

All studies must disclose on these points even when the disclosure is negative.

Sample size	No statistical methods were used to determine the sample size in this study. The sample sizes are clearly described in the figure legends. The sample size were determined by allowable error size, accuracy, resources, and need for statistical analysis (generally n>=3 throughout all the studies of this paper).
Data exclusions	There were no data exclusions.
Replication	The experiments that led to the conclusions of this study were independently and successfully reproduced for 2 times. For the benchmark formulation, experiments were independently and successfully reproduced for 5 times.
Randomization	For the animal experiment described in this study, mice were randomized prior to injection of lipid nanoparticles. There were no randomization practices in other experiments (CICS characterizations of mRNA LNPs) as there was no covariate in the study design.
Blinding	There were no blinding practices in the study design, because our study (both the animal study and CICS characterizations of mRNA LNPs) did not use any quantitative assessments that depend on objective evaluations or measurements.

Reporting for specific materials, systems and methods

We require information from authors about some types of materials, experimental systems and methods used in many studies. Here, indicate whether each material, system or method listed is relevant to your study. If you are not sure if a list item applies to your research, read the appropriate section before selecting a response.

Materials & experimental systems

n/a	Involved in the study	
x		Antibodies
x		Eukaryotic cell lines
x		Palaeontology and archaeology
	x	Animals and other organisms
x		Human research participants
x		Clinical data
x		Dual use research of concern

Methods

n/a	Involved in the study	
x		ChIP-seq
x		Flow cytometry
x		MRI-based neuroimaging

Animals and other organisms

Policy information about studies involving animals; ARRIVE guidelines recommended for reporting animal research

Laboratory animals	The study used Balb/cJ mice, female, 8 weeks in age.
Wild animals	The study did not involve wild animals.
Field-collected samples	The study did not involve field-collected samples.
Ethics oversight	Johns Hopkins University Animal Care and Use Committee (JHU ACUC) approved the animal protocol (intraveneous injection of lipid nanoparticles followed by live-animal imaging and organ harvest) used in this study. Protocol number: MO20E63.

Note that full information on the approval of the study protocol must also be provided in the manuscript.
